# Daptomycin eosinophilic pneumonia, a systematic review of the literature and case series

**DOI:** 10.1007/s15010-024-02349-z

**Published:** 2024-07-19

**Authors:** Anna Gidari, Carlo Pallotto, Daniela Francisci

**Affiliations:** Department of Medicine, Clinic of Infectious Diseases, “Santa Maria della Misericordia” Hospital, University of Perugia, Piazzale Lucio Severi 1, Perugia, 06132 Italy

**Keywords:** Daptomycin, Eosinophilic pneumonia, ARDS, Adverse event

## Abstract

**Purpose:**

Daptomycin-induced eosinophilic pneumonia (DIEP) is a rare yet severe adverse event that requires rapid recognition and management. Diagnosing a definite case is challenging and involves meeting the American Thoracic Society (ATS) criteria, although alternative criteria have been suggested. This study aims to conduct a systematic review of literature and includes a case series.

**Methods:**

Six cases of DIEP identified at Perugia Hospital, Perugia, Italy have been described. A systematic review was carried out adhering to the Preferred Reporting Items for Systematic Reviews and Meta-Analyses (PRISMA) Statement guidelines.

**Results:**

a total of 74 cases of DIEP were analysed. Using ATS clinical criteria, 15 were classified as definite (20.3%), 54 as probable (73.0%), and 5 as possible (6.8%). Phillips criteria and the Lyon Algorithm identified 43/74 (58.2%) and 64/67 (95.5%) cases as definite, respectively. Bronchoalveolar lavage (BAL) was performed in 43 cases, revealing an average eosinophil count of 28.6% (SD 24.4). Radiological findings highlighted recurring features like bilateral opacities (68.1%), ground-glass opacities (41.7%), patchy infiltrates (30.6%), and peripheral predominance (19.4%). Upon suspicion, daptomycin was discontinued; 20 cases required no additional treatment, 38 received corticosteroids, and 12 received both corticosteroids and antibiotics. Recovery rates were high across all treatment types (≥ 73.7%). Most reports described rapid improvement post-withdrawal (within 96 h).

**Conclusions:**

DIEP is a rare, fast-progressing condition where early diagnosis and prompt treatment are vital. Diagnosis relies on clinical, laboratory, and radiological evaluations. Stopping daptomycin is essential, with corticosteroids often necessary. Further research is needed to enhance diagnostic accuracy for this disease.

## Introduction

Daptomycin is an antibiotic of the class of lipopeptides. Its spectrum of activity includes most gram-positive bacteria. It is widely used in clinical practice and, being administrable once daily, also for long-term therapy in patients discharged from the hospital [1]. Despite its higher cost compared to other antibiotics, its preference is justified by favourable handling characteristics, low resistance development, and a strong safety profile [1–3]. Notably, daptomycin combined with rifampicin has demonstrated effectiveness in treating complex device-related infections due to its biofilm activity [1, 4].

Daptomycin is generally well-tolerated, yet like all medications, it carries potential adverse effects. One of the most severe is eosinophilic pneumonia, specifically daptomycin-induced eosinophilic pneumonia (DIEP), as documented in the literature. The exact mechanism behind daptomycin’s pulmonary toxicity remains unclear, but several theories have been proposed. Daptomycin is quickly inactivated by pulmonary surfactant. The drug is then absorbed by alveolar macrophages which present it as an antigen to T helper cells. These cells subsequently produce interleukin-5. Alongside eotaxin, which is released by endothelial cells, interleukin-5 leads to the recruitment of eosinophils into the alveolar spaces. This sequence of events triggers an inflammatory cascade that causes acute epithelial damage [5, 6]. Daptomycin can induce eosinophilic pneumonia causing dyspnea, hypoxemia and respiratory failure [7]. The literature provides limited and unclear information about this disease; however, given its severity and rapid progression, this type of pneumonia requires prompt diagnosis, as well as more specific diagnostic criteria and treatment approaches.

This study aims to present a series of cases collected between January 2019 and June 2020 in Santa Maria della Misericordia Hospital, Perugia, Italy. We also performed a systematic review of the available literature.

## Case series

### Case 1

P. U., a 69-year-old man, was admitted on February 16, 2019, diagnosed with Methicillin-Sensitive *Staphylococcus aureus* (MSSA) endocarditis affecting his native aortic valve, accompanied by multiple emboli and abscess formations. His medical history included two prior surgeries—a discectomy and a hip prosthesis implant—though no significant pathologies or known allergies were reported.

Upon admission, a comprehensive body CT scan revealed multiple findings: retroperitoneal abscess collections near the retrocaval area and within both psoas muscles, a cortical abscess in the right kidney, and several non-colliquated external iliac lymph nodes. In the upper and posterior mediastinum, additional abscess collections were identified, with the most significant adjacent to the thoracic esophagus, below the azygos vein, and behind the subclavian artery. Another abscess was located near the right scapular glenoid. Additionally, a septic embolus was noted in the dorsal segment of the right upper lobe.

Given these findings, the patient urgently underwent thoracoscopy for the placement of pleural and abscess drains. The procedure, however, was complicated by the development of a pneumothorax.The chosen treatment regimen began with oxacillin (3 gm IV q4h) and clindamycin (600 mg IV q8h). The treatment faced challenges, including delayed blood culture negativity extending beyond seven days and ongoing fever. As a result, on March 8, clindamycin was substituted with daptomycin (10 mg/kg per day) and rifampicin (600 mg IV q24h), while continuing oxacillin. Since the modification did not yield significant improvements, daptomycin was discontinued after a week. Despite the complex clinical situation, the patient did not experience respiratory failure during the initial three weeks. On March 28, daptomycin at a dosage of 10 mg/kg per day was reintroduced to his treatment. Eight days later, the patient developed symptoms including fever and respiratory failure characterized by hypoxemia and bronchospasm. Laboratory tests indicated elevated white blood cell count (12,560/mmc) and peripheral eosinophil count (690.8/mmc, 5.5%), along with a significant increase in C-Reactive Protein (CRP, 21.6 mg/dl, normal < 0.5 mg/dl). High-resolution computed tomography (HRCT) of the chest revealed bilateral patchy pneumonic infiltrates and bilateral pleural effusion. Eosinophilic pneumonia was suspected, leading to the immediate cessation of daptomycin and initiation of corticosteroid therapy. The patient’s respiratory function improved significantly within 24 h, and inflammatory markers gradually decreased. He was discharged on May 17, 2019, without the need for oxygen or ongoing corticosteroid treatment. A follow-up chest radiograph five weeks later confirmed the complete resolution of the radiological findings. This case was deemed probable according to ATS criteria and definite according to the Lyon algorithm, although it did not meet the Phillips Criteria [8–10].

### Case 2

R. C., a 67-year-old man, was admitted to the post-cardiac surgery intensive care unit on April 9, 2019, following the replacement of his aortic and mitral valves with bio-prosthetic valves. His medical background included chronic kidney disease, chronic obstructive pulmonary disease (COPD), hemosiderosis, non-Hodgkin lymphoma, and allergies to amoxicillin/clavulanate and ceftriaxone.

Following the valve replacement surgery, a sample from the mitral valve was analyzed in the microbiology lab and tested positive for Methicillin-resistant *S. epidermidis* (MRSE). Based on the sensitivity profile, he was started on daptomycin at a dosage of 10 mg/kg. After 22 days on daptomycin, he presented with a high fever and respiratory failure. Laboratory tests showed elevated white blood cell count (19,790/mmc), peripheral eosinophil count (1,504/mmc, 7.6%), and CRP (28 mg/dl, normal < 0.5 mg/dl). The HRCT of the chest revealed bilateral patchy reticular interstitial patterns with ground glass opacities and septal thickening, along with left pleural effusion. Bronchoalveolar lavage identified 20.5% eosinophils, while tests for bacterial and *Pneumocystis jirovecii* infections returned negative results.

Given the suspicion of eosinophilic pneumonia, daptomycin was discontinued and corticosteroid therapy initiated. The patient’s respiratory function improved significantly within 96 h, and inflammatory markers decreased steadily. He was discharged without the need for supplemental oxygen or continued corticosteroid treatment. A follow-up chest radiograph two months later confirmed the complete resolution of the radiological findings. This case was classified as probable according to ATS criteria and definite according to both the Lyon algorithm and Phillips Criteria [8–10].

### Case 3

P. F., a 77-year-old man, was admitted to the hospital for osteomyelitis on March 27, 2019. His medical history included chronic kidney disease, COPD, and an allergy to iodinated contrast media. Upon admission, he was placed on empirical treatment with daptomycin (7 mg/kg per day), ceftazidime (2 gm IV q8h), and ciprofloxacin (400 mg IV q12h). Four days into the treatment, he developed high fever and respiratory failure. Laboratory tests showed an increase in white blood cell count (9,840/mmc), peripheral eosinophil count (1,043/mmc, 10.6%), and CRP (7.9 mg/dl, normal < 0.5 mg/dl). Corticosteroid therapy was initiated immediately. The HRCT of the chest revealed bilateral patchy reticular interstitial patterns with ground glass opacities, predominantly in the upper regions. Bronchoalveolar lavage performed two days post-corticosteroid therapy showed only 0.3% eosinophils, and all microbiological tests for bacteria, viruses, and fungi returned negative results. Given the suspicion of eosinophilic pneumonia, daptomycin was discontinued. The patient’s respiratory function improved significantly within 36 h, and his inflammatory markers decreased steadily. He was discharged without the need for supplemental oxygen but continued on low-dose corticosteroid therapy. Follow-up chest radiography two months later confirmed the complete resolution of the radiological findings. This case has been classified as probable according to ATS criteria and definite according to both the Lyon algorithm and Phillips Criteria [8–10].

### Case 4

T. M., a 75-year-old outpatient, was receiving daptomycin (7.73 mg/kg/die) for an early prosthetic knee infection. His medical background included COPD, high blood pressure, benign prostatic hyperplasia (BPH), and gastroesophageal reflux disease, with no known kidney injury or history of allergies. After 34 days on daptomycin, he exhibited symptoms of high fever and respiratory failure and was admitted to the hospital on July 22, 2019. Laboratory tests showed a normal white blood cell count (8,500/mmc) but an elevated peripheral eosinophil count (1,122/mmc, 13.2%) and CRP (7.9 mg/dl, normal < 0.5 mg/dl). HRCT scans of the chest revealed a bilateral patchy reticular interstitial pattern with ground glass opacities and septal thickening. Bronchoalveolar lavage was not conducted. Given the suspicion of eosinophilic pneumonia, daptomycin was discontinued, and treatment with corticosteroids and a new antibiotic regimen began. The patient’s respiratory function improved significantly within 48 h, and his inflammatory markers gradually decreased. He was discharged without the need for oxygen or ongoing corticosteroid treatment. This case was classified as probable according to ATS criteria and definite according to the Lyon algorithm, though it did not meet the Phillips Criteria [8–10].

### Case 5

C. F., a 67-year-old man, was admitted to the post-cardiac surgery intensive care unit on September 27, 2019, following an aortic-coronary bypass operation. His medical history included ischemic cardiomyopathy, type 2 diabetes, hypertension, chronic kidney disease, COPD, and dyslipidemia, but no known allergies. During his stay, he developed acute kidney failure and a MSSA surgical wound and bloodstream infection. Based on the antibiogram results, treatment with daptomycin (11.67 mg/kg) commenced on October 29, 2019.

Seventeen days into treatment, he presented with high fever and Acute Respiratory Distress Syndrome (ARDS). Laboratory results indicated a normal white blood cell count (6,330/mmc) but an increased peripheral eosinophil count (367/mmc, 5.8%) and CRP (7.3 mg/dl, normal < 0.5 mg/dl). HRCT scans displayed a bilateral patchy reticular interstitial pattern with ground glass opacities and septal thickening, along with bilateral pleural effusion. Bronchoalveolar lavage revealed an eosinophil percentage of 11%, and all microbiological tests for bacteria, fungi, and *Pneumocystis jirovecii* returned negative results. Suspecting eosinophilic pneumonia, daptomycin was replaced with ceftobiprole, which showed limited effectiveness. Corticosteroid therapy commenced ten days later, leading to rapid improvement in respiratory function and a gradual reduction in inflammatory markers. Corticosteroids and oxygen therapy were eventually discontinued. A chest radiograph taken five weeks later confirmed the complete resolution of the radiological abnormalities. This case was classified as probable according to ATS criteria and definite according to the Lyon algorithm, though it did not fulfill the Phillips Criteria [8–10].

### Case 6

On June 1st, 2019, B. S., a 43-year-old woman with metabolic syndrome, uncontrolled type 2 diabetes, and a history of osteomyelitis, was admitted due to severe pain in her right leg and foot. Physical examination showed crepitation in her leg and a fever. Radiography revealed pulmonary blurring, furthermore, X-rays identified air within the superficial cutaneous layers of her leg and discontinuity in the cortical bone. Early signs of ischemia in her leg led to the disarticulation of the fifth toe and multiple surgical incisions that drained purulent material. She initiated antibiotic treatment with meropenem and teicoplanin. She was diagnosed with sepsis and necrotizing fasciitis after blood, skin biopsy, and swab cultures isolated several types of gram-positive cocci, including Streptococci and Bacteroides. On June 3rd, her antibiotic regimen was adjusted, replacing teicoplanin with ceftaroline and daptomycin due to the rapid spread of infection on healthy skin and emerging respiratory distress, prompting urgent surgical intervention. Her right leg was amputated. Echocardiography ruled out endocarditis.

As healing was poor, tigecycline was added to target a multi-resistant Acinetobacter baumannii identified in a rectal swab. Despite these measures, a higher-level amputation was necessary. On June 23rd, daptomycin was discontinued, lightening the antibiotic load. The following day, she experienced a rapid decline in respiratory function and became dyspnoic; CT scans showed patchy areas, and peripheral eosinophilia (16%) raised suspicions of DIEP, although bronchoalveolar lavage was not performed due to her severe respiratory distress.

Her respiratory condition gradually improved after all antibiotic treatments were halted and replaced with amoxicillin/clavulanate. She was discharged on July 10th in stable condition, with a clean wound and low-dose oxygen therapy. This case meets the criteria for a probable diagnosis according to ATS guidelines, and definite according to both the Lyon algorithm and Phillips Criteria [8–10].

## Systematic review of literature

### Materials and methods

We conducted a systematic review of literature following Preferred Reporting Items for Systematic Reviews and MetaAnalyses (PRISMA) Statement protocol [11].

A systematic search on PubMed, Google Scholar, Directory of Open Access Journal and Cochrane Database of Systematic Reviews (DOAJ) was performed from inception to the end of September 2023. We used the following search strategy: every search included the terms “eosinophilic”, “pneumonia” and “daptomycin”. Each article was analyzed to establish if reached eligibility criteria. The inclusion criteria were as follows: English, Italian or Spanish language, pertinence on the issue, and articles that reported sufficient patient information such as age, sex, clinical and radiological presentation, treatment and outcome.

The exclusion criteria were as follows: languages different from those mentioned above; abstract and posters from conference proceedings because they did not pass under literature review. All cases have been evaluated according to American Thoracic Society (ATS) criteria, Phillips criteria and Lyon algorithm [8–10].

Data were summarized using descriptive statistical analysis where possible. Descriptive characteristics were summarized using numbers and percentages for categorical variables, and either means with standard deviations (SD) or medians with interquartile ranges (IQR) for numerical variables, as appropriate. The Kolmogorov-Smirnov test was employed to determine the distribution of continuous variables.

Additionally, the Chi-square test was used to assess whether different treatments were associated with recovery or improvement outcomes. A p-value of < 0.05 was considered statistically significant.

## Results

A total of 115 results were found on Google Scholar, 71 on Pubmed and 17 on DOAJ, among these 44 papers matched the eligibility criteria. There were 32 case reports, 7 case series, 3 case reports plus review and 2 case series plus review, with a total of 68 cases. We analyzed data also including our five presented cases, with a total of 74. Among these, applying clinical criteria from ATS [8], we identified 15 definite cases (20.3%), 54 probable cases (73.0%), 5 possible cases (6.8%), and no unlikely cases. Patients’ characteristics are summarized in Table 1.

**Table 1:** A systematic review of literature: clinical characteristics of cases with suspected daptomycin induced eosinophilic pneumonia (DIEP).

	*N* 74
Classification according to the American Thoracic Society Committee (N 74):	
Definite, N (%) Probable, N (%) Possible, N (%) Unlikely, N (%)	15 (20.3) 54 (73.0) 5 (6.80) 0 (0)
Phillips criteria (N 74): definite, N (%)	43 (58.1)
Lyon criteria (N 67):	
Definite, N (%) Probable, N (%) Unlikely, N (%)	64 (95.5) 3 (4.5) 0 (0)
Age (N 73): median, years (IQR) [range]	67.0 (60.0–76.0) [26–92]
Sex (N 73): male N (%)	58 (79.4)
Previous allergic reactions (N 21), N (%)	9 (42.9)
Chronic lung disease (N 55), N (%)	12 (21.8)
Acute or chronic renal impairment (N 53)	18 (34.0)
Daptomycin dose (N 52), median, mg/kg/die (IQR) [range]	6.0 (6–7.9) [4.0–11.7]
Duration of daptomycin therapy (N 69), median, days (IQR) [range]	19.0 (12–24) [2–54]
Symptoms at presentation:	
Fever, N (%) (N 70) Respiratory symptoms and/or hypoxemia, N (%) (74)	49 (70.0) 67 (90.5)
Peripheral white blood cells (N 47)	
Mean, /mmc (STD) [range]	12521.0 (5383.0) [210–26,360]
Peripheral eosinophil	
Median, /mmc (IQR) [range] (N 48) % Median, (IQR) [range] (N 52)	936.4 (568.5 – 1641.1) [0-4620.0] 7.7 (5.5–13.4) [0–30.0]
Eosinophils in bronchoalveolar lavage (N 43):	
Median, N (%) (IQR) [range] > 25%, N (%)	20.5 (10.5–41.0) [0–98.0] 21 (48.8)
Radiological findings (N72):	
Bilateral opacities, N (%) Ground-glass opacities, N (%) Patchy infiltrates, N (%) Peripheral predominance, N (%)	49 (68.1) 30 (41.7) 22 (30.6) 14 (19.4)
Treatment (N 74):	
DAP withdrawal, N (%) Recovery, N (%) DAP withdrawal plus corticosteroid, N (%) Recovery, N (%) DAP withdrawal plus corticosteroid plus antibiotic therapy, N (%) Recovery, N (%) DAP withdrawal plus antibiotic therapy, N (%) Recovery, N (%)	20 (27.0) 17 (85.0) 38 (51.4) 28 (73.7) 12 (16.2) 10 (83.3) 4 (5.4) 3 (75.0)
Outcome (N 74):	
Recovery, N (%) Improvement, N (%) Death, N (%)	58 (78.4) 14 (18.9) 2 (2.7)
Timing of improvement (N 46):	
Median, days (interquartile range) After 24 h, N (%) After 48 h, N (%) After 72 h, N (%) After 96 h, N (%) Total of patients improved after 96 h, N (%)	2.0 (1.0-3.8) 13 (28.3) 13 (28.3) 8 (17.4) 6 (13.0) 40 (87.0)

*Footnotes*: N, number of cases; IQR, interquartile range; STD, standard deviation; DAP, daptomycin

Of note, six cases did not match the criteria for definite cases because the authors did not report if the patient had or not fever. Applying criteria proposed by Phillips and colleagues [9], 43 (58.2%) cases are definite for DIEP. According to the Lyon algorithm, 64/67 (95.5%) cases are classified as definite. Of note, about 7 cases data were not enough to apply the Lyon algorithm [10].

The median age was 67.0 (interquartile range, IQR, 60.0–76.0, range 26–92) and 58 (79.4%) cases were male. The median daptomycin dose was 6.0 mg/kg/die (IQR 6-7.9, range 4–11.7) and the eosinophilic pneumonia symptoms appeared after a median of 19.0 days (IQR 12–24) of treatment. The most common initial symptoms were fever (49 cases, 70%) and respiratory symptoms (67 cases, 90.5%) such as dyspnea. For 52 cases, the value of peripheral eosinophils has been reported (in absolute number or percentage), among these, eosinophilia was diagnosed in 45 patients (86.5%). The bronchoalveolar lavage (BAL) was performed in 46 cases but eosinophils count was available for 43 cases with a median of eosinophils of 20.5% (IQR 10.5–20.5, range 0–98.0). Of note, the only patient with 0% eosinophils on BAL and peripheral blood count was a leukopenic woman with hematologic disease [12]. A lung biopsy was performed in eight cases and in six of them eosinophilic pneumonia was confirmed.

Radiological findings (available for 72 cases) were various, but some findings recurred such as bilateral opacities (49 cases, 68.1%), ground-glass opacities (30 cases, 41.7%), patchy infiltrates (22 cases, 30.6%) and peripheral predominance (14 cases, 19.4%).

In all cases, when eosinophilic pneumonia was suspected, daptomycin was withdrawn and in 20 cases (27.0%) no other therapy was added. In 38 cases (51.4%), the daptomycin withdrawal was associated only with corticosteroid therapy and in 12 cases (16.2%) with also antibiotic therapy. In 4 cases (5.4%), corticosteroid was not necessary but antibiotic therapy was added. As shown in Table 1, for each kind of treatment we found a good percentage of recovery (≥ 73.7%). Among the different treatments, none were significantly associated with a higher recovery rate (*p* > 0.05). The outcome was favorable for most of the patients with a recovery of 78.4% and improvement of 18.9%. Two patients (2.7%) died. Furthermore, a rapid improvement after daptomycin withdrawal has been described in most of the reports. The time of improvement was available for 46 cases and most of them (40 cases, 87.0%) significantly improved after 96 h. The improvement has been especially described as oxygen support reduction/suspension.

Specific data from every case included in the literature are resumed in Table 2.

**Table 2:** Summary of 74 cases of presumed daptomycin-induced eosinophilic pneumonia

Case	Classification (ATS)	Phillips criteria	Lyon algorithm	Age	Sex	Indication	Dose of daptomycin (mg/kg/die) duration (days)	Fever	Respiratory symptoms and/or hypoxemia	BAL eosinophils (%); lung biopsy	Peripheral eosinophils /mmc; %	Radiological findings	Treatment	Outcome	Timing of recovery/improvement (days)
Hayes [7] (2007)	Definite	Yes	Definite	60	M	MSSA endocarditis	NA; 14	Yes	Yes	26; Not performed	NA; NA	bilateral patchy area of consolidations in a peripheral distribution	Withdrawal plus Corticosteroid	Recovery	improved after 24 h
Cobb [13] (2007)	Possible	No	Definite	84	M	MRSA Septic arthritis	4; 47	No	No	Not performed; Organized pneumonia and some eosinophilic infiltrate	324; 4	bilateral, patchy nodules.	Withdrawal	Recovery	Partial resolution at 2 weeks
Scinde [14] (2008)	Possible	Yes	NA	54	M	MRSA post-surgical infection	NA; 14	Yes	Yes	Not performed; Organized pneumonia with eosinophilic infiltrate	NA; NA	bilateral patchy infiltrates and peripheral opacities	Withdrawal plus Corticosteroid	Recovery	improved after 24 h/recovery at 4 weeks
Kakish [15] (2008)	Definite	Yes	Definite	65	M	MRSA osteomyelitis and epidural abscess	6; 14	Yes	Yes	33; Not performed	602; 7	diffuse bilateral airspace sparing the bases disease with some peripheral predominance, and small bilateral pleural effusions	Withdrawal plus Corticosteroid	Recovery	improved after 72 h/CT normalized after 3 months
Lal [16] (2010)	Probable	Yes	Definite	82	M	S. mitis prosthetic joint infection	NA; 21	Yes	Yes	14; Not performed	1639; 11	patchy bilateral ground glass infiltrates	Withdrawal plus Corticosteroid	Improvement	Asymptomatic after 5 days
Lal [16] (2010)	Definite	Yes	Definite	87	M	Prosthetic joint infection	NA; 21	Yes	Yes	40; Not performed	1306.4; 9.2	patchy bilateral ground glass infiltrates	Withdrawal plus Corticosteroid	Recovery	Improved after 24 h
Miller [17] (2010)	Definite	Yes	Definite	60	M	MSSA prosthetic joint infection	6; 12	Yes	Yes	81; Acute fibrinous and organizing pneumonia	4620; 21	bilateral scattered ground glass opacities and peripheral consolidation	Withdrawal plus Corticosteroid	Recovery	Asymptomatic after 7 days
Miller [17] (2010)	Probable	No	Definite	60	M	MRSA Septic arthritis	6; 12	Yes	Yes	Not performed; Not performed	990; 9	patchy peripheral nodular and ground glass infiltrates	Withdrawal plus Corticosteroid	Recovery	Asymptomatic after 5 days
Miller [17] (2010)	Probable	Yes	Definite	83	M	Diskitis	6; 10	Yes	No	Not performed; Acute organizing pneumonia, eosinophilia	936; 9	diffuse ground glass and reticular opacities with mild septal thickening with associated small bilateral pleural effusions	Withdrawal	Recovery	Asymptomatic after 6 days/ recovery after 4 weeks
Kalogeropoulos [18] (2011)	Definite	Yes	Definite	78	M	Empirical treatment of Endocarditis	8; 10	Yes	Yes	27.5; Not performed	229.31; 2.3	patchy bilateral infiltrates with ground glass peripheral opacities and bilateral pleural effusions	Withdrawal plus Corticosteroid	Recovery	Fever resolved after 24 h from withdrawal/normal HRTC at 4 weeks
Rether [19] (2011)	Definite	Yes	Definite	69	M	spondylodiscitis with lumbar epidural and bilateral psoas abscesses due to Enterococcus faecium	6; 21	Yes	Yes	30; Not performed	NA; Normal	extensive bilateral patchy infiltrates involving the right lower lobe and entire left lung	Withdrawal plus Corticosteroid	Recovery	Improved after 24 h
Kim [8] (2012)	Definite	No	Definite	63	F	MSSA spinal osteomyelitis	6; 21	Yes	Yes	65; Not performed	NA; 10.5	NA	Withdrawal plus Corticosteroid	Recovery	NA
Kim [8] (2012)	Definite	Yes	Definite	64	M	S. aureus osteomyelitis with bacteremia	5.7; 28	Yes	Yes	44; Not performed	NA; NA	peripheral pulmonary infiltrates	Withdrawal	Recovery	NA
Kim [8] (2012)	Probable	Yes	Definite	79	M	Enterococcal endocarditis	6; 42	Yes	Yes	11; Eosinophilic pneumonitis	NA; Not evaluable	extended ground glass opacities	Withdrawal plus Corticosteroid	improvement	NA
Kim [8] (2012)	Probable	Yes	NA	26	M	MRSA bacteraemia	7.35; 10	No	Yes	Not quantified; Not performed	NA; NA	new pulmonary infiltrates	Withdrawal	improvement	NA
Kim [8] (2012)	Probable	Yes	Definite	43	M	MRSA osteomyelitis	6; 10.5	No	Yes	Not performed; Not performed	NA; 6.6	bilateral infiltrates	Withdrawal	improvement	NA
Kim [8] (2012)	Probable	No	NA	66	M	MSSA bacteraemia	6; 7	No	Yes	Not quantified; Not performed	NA; NA	NA	Withdrawal plus Corticosteroid	Recovery	NA
Kim [8] (2012)	Probable	No	Definite	71	M	MRSA diabetic foot infection	4; 54	No	Yes	Not performed; Not performed	NA; 8	bilateral interstitial opacities	Withdrawal	improvement	NA
Kim [8] (2012)	Probable	No	NA	77	F	Enterococcal bacteraemia	5; 7	No	Yes	Not performed; Not performed	NA; NA	pneumonitis	Withdrawal plus Corticosteroid	improvement	NA
Kim [8] (2012)	Probable	Yes	Definite	67	M	MRSA endocarditis	6; 30	No	Yes	9; Not performed	NA; NA	bilateral pulmonary infiltrates	Withdrawal plus Corticosteroid	improvement	NA
Kim [8] (2012)	Probable	No	NA	73	M	MRSA prosthetic joint infection	5; 26	Yes	Yes	Not performed; Not performed	NA; NA	bilateral ground glass infiltrates	Withdrawal plus Corticosteroid	Recovery	NA
Kim [8] (2012)	Probable	Yes	NA	81	F	MRSA paraspinal abscess	6; 11	No	Yes	2; Not performed	NA; NA	bilateral mid-lung infiltrates	Withdrawal plus Corticosteroid	improvement	NA
Phillips [9] (2013)	Probable	Yes	Definite	48	M	MSSA osteomyelitis	6; 21	Yes	Yes	17; Not performed	1554; 10.5	patchy bilateral airspace opacities	Withdrawal plus Corticosteroid	Recovery	Improved after 24 h after corticosteroid initiation
Phillips [9] (2013)	Probable	Yes	Definite	28	M	MRSA osteomyelitis	6; 28	NA	Yes	74; Not performed	2926; 19	diffuse multilobar infiltrates	Withdrawal plus Corticosteroid plus Antibiotic therapy.	Recovery	Asymptomatic after one week of corticosteroid therapy
Yusuf [20] (2014)	Probable	No	Definite	64	M	MRSE prosthetic joint infection	10; 28	Yes	No	47; Not performed	730; 5	diffuse bilateral patchy ground glass opacities	Withdrawal	Recovery	Afebrile after 24 h of withdrawal
Yusuf [20] (2014)	Probable	Yes	Definite	61	M	MRSE prosthetic joint infection	10; 14	Yes	Yes	3; Not performed	1230; 15	bilateral ground glass consolidation with bilateral pleural effusion	Withdrawal plus Corticosteroid plus Antibiotic therapy.	Recovery	Recovery after 24 h
Patel [5] (2014)	Probable	Yes	Definite	61	F	Osteomyelitis	NA; 7	NA	Yes	30; Not performed	2415; 15	bilateral pleural effusion and bilateral patchy infiltrates	Withdrawal plus Corticosteroid	Recovery	Extubated after 72 h
Hagiya [21] (2015)	Probable	No	Definite	34	M	MSSA endocarditis	10; 2	Yes	Yes	Not performed; Not performed	510; NA	peripheral consolidation	Withdrawal	Recovery	CT findings disappeared after 6 weeks
Chiu [22] (2015)	Probable	Yes	Definite	77	M	S. aureus and VRE osteomyelitis	6; 42	No	Yes	18; Not performed	708; 3.1	bilateral airspace disease	Withdrawal plus Corticosteroid plus Antibiotic therapy.	Recovery	Improved after 60 h
Chiu [22] (2015)	Possible	No	Probable	74	F	Infected hip reconstruction	6; 3	Yes	Yes	Not performed; Not performed	NA; Normal	bilateral airspace disease	Withdrawal plus Corticosteroid	Recovery	Improved after 24 h: weaned off NIV and placed on Nasal cannula
Wojtaszczyk [23] (2015)	Definite	Yes	Definite	76	M	MRSA Septic arthritis	NA; 14	Yes	Yes	54; Not performed	235.2; 2.1	bilateral ground glass opacities and patchy areas of consolidation in a peripheral distribution	Withdrawal plus Corticosteroid	improvement	Hypoxemia resolved after 72 h
Roux [24] (2015)	Probable	Yes	Definite	67	M	MSSA prosthetic joint infection	6; 17	NA	Yes	10; Not performed	2600; NA	bilateral diffuse alveolar and interstitial opacities	Withdrawal plus Corticosteroid	Recovery	Improved after 96 h from withdrawal/Rx normalized after 3 weeks
Montenegro [25] (2015)	Definite	Yes	Definite	54	M	MRSA Osteomyelitis and endocarditis	6; 28	Yes	Yes	30; Not performed	NA; 15	bilateral patchy infiltrates	Withdrawal	Recovery	Improved after 72 h/Recovery after 7 days
Akcaer [26] (2016)	Probable	No	Definite	60	M	MSSA subcutaneous abscess	5; 24	No	Yes	Not performed; Not performed	2288.4; 14.1	right pleural effusions, bilateral tree-in-bud pattern, bilateral scattered ground glass opacities	Withdrawal	Recovery	Clinical resolution at 72 h/cured after 9 days
Hatipoglu [27] (2016)	Possible	No	Probable	67	F	MRSA Diabetic foot infection	NA; 23	Yes	Yes	Not performed; Not performed	160; 1.3	right lobe infiltration	Withdrawal plus antibiotic therapy	Recovery	Improved after 72 h/Recovery after 8 days
Hirai [28] (2016)	Probable	No	Definite	64	M	S. aureus prosthetic joint infection	8; 20	Yes	No	14; Not performed	847; 7.7	bilateral multiple reticulonodular infiltrates and ground glass opacities on the subpleural region	Withdrawal	Recovery	Afebrile after 72 h
Hirai [28] (2016)	Probable	No	Definite	74	M	S. caprae septic arthritis	5.8; 24	Yes	No	Not performed; Not performed	936.9; 9.1	bilateral multiple reticulonodular infiltrates on the subpleural region	Withdrawal	Recovery	Afebrile after 48 h
Hirai [28] (2016)	Probable	Yes	Definite	77	M	Methicillin-resistant S. capitis prosthetic joint infection	5.3; 14	Yes	Yes	19,2 Not performed	330; 6.6	bilateral multiple infiltrates	Withdrawal	Recovery	Asymptomatic after 48 h
Hirai [28] (2016)	Probable	No	Definite	61	M	MRSA foot ulcer	6; 24	Yes	Yes	Not performed; Not performed	888; 7.4	bilateral multiple reticulonodular infiltrates and ground glass opacities on the subpleural region	Withdrawal	Recovery	Improved after 96 h
Hirai [28] (2016)	Probable	No	Definite	67	M	MRSA spondylitis	5.8; 52	Yes	No	Not performed; Not performed	310; 5.2	bilateral multiple reticulonodular infiltrates and ground glass opacities on the subpleural region	Withdrawal	Recovery	Afebrile after 48 h
Hirai [28] (2016)	Probable	No	Definite	69	M	MRSA surgical site infection	5.2; 5	Yes	No	36,5; Not performed	1120; 13.5	bilateral diffuse infiltrates in the subpleural region	Withdrawal	Recovery	Asymptomatic after 96 h
Goyal [29] (2016)	Probable	Yes	Definite	85	M	Osteomyelitis	NA; 21	No	Yes	75; Not performed	1300; NA	Right upper lung and left basilar opacities	Withdrawal plus Corticosteroid	Recovery	No timing
Nickerson [30] (2017)	Probable	Yes	Definite	70	F	MRSA osteomyelitis	6; 14	No	Yes	5; Not performed	1647.2; 11.6	diffuse bilateral infiltrates with interstitial predominance and some alveolar and ground glass components	Withdrawal	Recovery	Improved after 48 h
Rachid [31] (2017)	Probable	Yes	Definite	64	M	MRSA groin graft infection	6; 42	Yes	Yes	Not performed; chronic inflammatory changes, dense fibrinous airspace exudates, area of organization and prominent eosinophils	999; 5.55	diffuse bilateral pulmonary infiltrates, mediastinal lymphadenopathy, and small bilateral pleural effusions	Withdrawal plus Corticosteroid	Recovery	Improved after 48 h/complete resolution of infiltrates after 7 days
Higashi [32] (2018)	Definite	Yes	Definite	53	M	MRSA foot ulcer, cellulitis and osteomyelitis	7; 24	Yes	Yes	69; Not performed	790.8; 3	diffuse bilateral patchy consolidations and multiple nodules in the peripheral regions	Withdrawal	Recovery	Rapidly improved
Raru [33] (2018)	Definite	Yes	Definite	68	M	S. epidermidis septic arthritis	NA; NA	Yes	Yes	28; Not performed	627; 11	interstitial and ground glass opacities	Withdrawal plus Corticosteroid	improvement	Improved at 4 weeks
Raru [33] (2018)	Probable	No	Definite	71	M	NA	NA; NA	Yes	Yes	Not performed; Not performed	416; 6.4	upper lobe bilateral infiltrates.	Withdrawal plus Corticosteroid	improvement	NA
Abelenda Alonso [34] (2018)	Definite	Yes	Definite	50	F	Post-surgical septic shock	5; 23	Yes	Yes	98; Not performed	4010; NA	bilateral interstitial pattern with a tarnished glass of peripheral predominance	Withdrawal plus Corticosteroid	Recovery	Improved after 48 h
Basnet [35] (2018)	Probable	No	Definite	89	M	CONS endocarditis	NA; 28	Yes	Yes	Not performed; Not performed	474; 6	bilateral lower ground glass opacification with interlobular septal thickening	Withdrawal plus Corticosteroid	improvement	Partial resolution of opacities at 2 weeks
Kumar [6] (2018)	Probable	Yes	Definite	65	M	Osteomyelitis	NA; 21	No	Yes	20.1; Not performed	1788; 14.9	bilateral pulmonary infiltrates	Withdrawal plus Corticosteroid	Recovery	Improved after 24–48 h/CT was negative after 3 weeks
Sharma [36] (2018)	Probable	Yes	Definite	52	F	Osteomyelitis	NA; 13	No	Yes	2.4; Not performed	2561.9; 13.7	bilateral peripheral ground glass opacity with septal thickening and areas of patchy opacities	Withdrawal plus Corticosteroid	Recovery	Improved after 12 h of corticosteroid therapy, daptomycin withdrawal was not enough/recovery after 2 weeks
Raza [37] (2019)	Probable	Yes	Definite	72	M	Osteomyelitis	NA; NA	NA	Yes	42; Not performed	3210; 30	bilateral ground glass opacities with right lung predominance	Withdrawal plus Corticosteroid	improvement	Improved in a few days/discharged on home oxygen
Storandt [38] (2020)	Definite	Yes	Definite	56	M	S. epidermidis spinal epidural abscess	6; 14	Yes	Yes	46; Not performed	NA; increased	bilateral patchy interstitial pneumonic infiltrates	Withdrawal plus Corticosteroid	Recovery	Radiological resolution after 7 weeks
Somoza-Cano [39] (2021)	Probable	No	Definite	79	M	Prosthetic joint infection	NA; 11	Yes	Yes	Not performed; Not performed	NA; increased	bilateral diffuse reticulonodular opacities in the lungs with peripheral predominance, areas of ground glass opacities, and nodularities.	Withdrawal plus Corticosteroid plus Antibiotic therapy.	Recovery	Rapidly improved, recovery after 1 month
Watts [40] (2021)	Probable	No	Definite	65	M	MRSA bloodstream infection and septic arthritis	8; 21	No	Yes	Not performed; Not performed	2480; 27	bilateral, multifocal consolidations, ground glass opacities	Withdrawal plus Corticosteroid	Recovery	Improved after 48 h
Raman [41] (2021)	Probable	No	Definite	71	M	MRSA endocarditis	NA; 14	No	Yes	Not performed; Not performed	963.9; 6.3	bilateral patchy airspace disease and ground glass infiltrate	Withdrawal plus Corticosteroid plus Antibiotic therapy.	death	Improved after 24 h
Portalatin [42] (2021)	Probable	No	Definite	53	M	MSSA bacteraemia and multiple small cervical paraspinous abscesses	NA; 10	Yes	Yes	Not performed; Not performed	790; 4.4	bilateral involvement of ground glass opacities predominantly in the periphery	Withdrawal plus Corticosteroid plus Antibiotic therapy.	Recovery	Improved after 48 h/Radiological fining disappeared after 4 weeks
Fernandez Gonzales [43] (2021)	Probable	No	Definite	92	M	MSSA bloodstream infection	10; 12	Yes	Yes	Not performed; Not performed	NA; NA	patchy consolidation areas associated with a reticular pattern in the middle lobe and lingula	Withdrawal plus Corticosteroid	Recovery	Improved after 24 h/recovery at 1 week
Fujii [44] (2022)	Probable	No	Definite	82	M	Empirical treatment of Endocarditis	8; 23	Yes	Yes	Not performed; Not performed	640; 7	bilateral ground glass opacities	Withdrawal plus Corticosteroid	Recovery	Improvement after 9 days/recovery after 2 weeks
Valaiyapathi [45] (2022)	Probable	Yes	NA	83	M	Prosthetic joint infection	NA; 28	No	Yes	Not quantified; Not performed	300; NA	bilateral extensive peripherally distributed ground glass opacities	Withdrawal plus Corticosteroid	Recovery	Recovery after 14 days
Eckhardt [46] (2022)	Probable	No	Definite	NA	NA	Prosthetic joint infection	NA; 28	No	Yes	10; eosinophils present but not increased	2420; 17	bilateral patchy ground glass opacities with associated consolidation and septal thickening	Withdrawal plus Corticosteroid plus Antibiotic therapy.	Recovery	Improved
Algayoum [47] (2022)	Probable	Yes	Definite	71	F	knee MRSA bursitis	NA; NA	Yes	Yes	6.5; alveolar spaces with collections of eosinophils and histiocytes surrounded by thickened alveolar septae with focal organization and focal hyaline membrane formation.	NA; NA	bilateral patchy ground glass opacities and septal thickening with peripheral predominance and some areas of septal thickening	Withdrawal plus Corticosteroid	Recovery	Improved after 48 h/ discharged after a few weeks
Gerts [48] (2022)	Definite	Yes	Definite	59	F	MRSA abscess	NA; 15	Yes	Yes	28; Not performed	NA; NA	bilateral airspace opacities with scattered subpleural nodular airspace opacities, mild bronchiectasis with peribronchial thickening in the lower lobes, small mediastinal lymph nodes with a prominent right hilar lymph node measuring 11–12 mm	Withdrawal plus antibiotic therapy	Recovery	Improved after withdrawal
Sharma [49] (2023)	Probable	Yes	Definite	65	M	Osteomyelitis	NA; 14	Yes	Yes	5; Not performed	NA; NA	extensive bilateral multifocal consolidations	Withdrawal plus Corticosteroid	Recovery	Improved after 96 h/discharged after 6 days
Zou [12] (2023)	Possible	No	Probable	44	F	Persistent MRSE bacteriemia (suspected endocarditis)	8; 19	Yes	Yes	0; Not performed	0; 0 (pancytopenia)	multifocal upper lobe-predominant bilateral ground glass opacities	Withdrawal plus antibiotic therapy	Recovery	Improve/discharged after 7 days / 2 weeks after daptomycin withdrawal, ground glass opacities decreased
Patel [50] (2023)	Probable	No	Definite	74	F	MRSA osteomyelitis	10; 13	No	Yes	Not performed; Not performed	350; 3.8	bilateral opacities and mediastinal adenopathy	Withdrawal plus Corticosteroid plus Antibiotic therapy.	Recovery	NA
Patel [50] (2023)	Probable	Yes	Definite	77	M	Osteomyelitis	6; 13	No	Yes	16; Not performed	600; 4.1	consolidating bilateral pulmonary opacities	Withdrawal plus Corticosteroid plus Antibiotic therapy.	death	Improved respiratory failure but death from cardiac arrest
Arcalas [51] (2023)	Probable	Yes	Definite	69	F	MRSA endocarditis	NA; NA	Yes	Yes	12; Not performed	1800; 7	bilateral patchy ground glass opacities and septal thickening with a crazy-paving. interstitial thickening	Withdrawal plus antibiotic therapy	improvement	Improved after 96 h from withdrawal/CT improved one month after discharge
Case 1 (P. U)	Probable	No	Definite	67	M	MSSA endocarditis	10; 8	Yes	Yes	Not performed; Not performed	690.8; 5.5	bilateral patchy pneumonic infiltrates	Withdrawal	Recovery	Improved after 24 h/discharged without oxygen/Radiological findings disappeared after 5 weeks
Case 2 (R. C.)	Probable	Yes	Definite	67	M	MRSE endocarditis	10; 22	Yes	Yes	20.5; Not performed	1504; 7.6	bilateral patchy reticular interstitial pattern with ground glass opacities and septal thickening. Left pleural effusion.	Withdrawal plus Corticosteroid	Recovery	Improved after 96 h/Radiological findings disappeared after two months, discharged without oxygen
Case 3 (P.F.)	Probable	Yes	Definite	77	M	Osteomyelitis	7; 4	Yes	Yes	0.3; Not performed	1043; 10.6	bilateral patchy reticular interstitial pattern with ground glass opacities and upper predominance	Withdrawal plus Corticosteroid plus Antibiotic therapy.	Recovery	Improved after 36 h/Radiological findings disappeared after 2 months
Case 4 (T. M.)	Probable	No	Definite	75	M	Prosthetic joint infection	7.7; 34	Yes	Yes	Not performed; Not performed	1122; 13.2	bilateral patchy reticular interstitial pattern with ground glass opacities and septal thickening	Withdrawal plus Corticosteroid plus Antibiotic therapy.	Recovery	Improved after 48 h/lost at follow up
Case 5 (C. F.)	Probable	Yes	Definite	66	M	MRSA bloodstream infection	11.7; 17	Yes	Yes	11; Not performed	367.1; 5.8	bilateral patchy reticular interstitial pattern with ground glass opacities and septal thickening, bilateral pleural effusion.	Withdrawal plus Corticosteroid plus Antibiotic therapy.	Recovery	Improved after corticosteroid therapy/Radiological resolution after 5 weeks
Case 6 (B. S.)	Probable	No	Definite	43	F	Gas gangrene	7.2; 22	No	Yes	Not performed; Not performed	3472.1; 29.6	bilateral patchy reticular interstitial pattern with ground glass opacities and septal thickening, bilateral pleural effusion.	Withdrawal	Recovery	Improved after 48 h

*Footnotes*: NA, not available; MSSA, Methicillin-Sensitive Staphylococcus Aureus; MRSA, Methicillin-Resistant Staphylococcus Aureus; CT, Computed Tomography; HRTC, High Resolution Computed Tomography; VRE, Vancomycin-Resistant Enterococcus; NIV, non-invasive ventilation; CONS coagulase-negative Staphylococci; MRSE, Methicillin-Resistant Staphylococcus Epidermidis

## Discussion

Daptomycin-induced eosinophilic pneumonia belongs to a diverse group of interstitial lung diseases, which are marked by peripheral blood eosinophilia, elevated eosinophils in BAL fluid, or eosinophilic infiltration of the lung parenchyma as confirmed by lung biopsy [52]. Various drugs, including antibiotics, non-steroidal anti-inflammatory drugs (NSAIDs), and cardiac anti-arrhythmic medications like amiodarone, have been linked to eosinophilic pneumonia [53]. Typically, patients with drug-induced eosinophilic pneumonia show a favourable response to steroid therapy and discontinuation of the offending drug [52].

Recent studies indicate a prevalence of DIEP between 4.8 and 15% among patients treated with daptomycin, increasing with age, particularly in those over 70 [54–56]. Factors such as male gender, longer treatment durations (over 21 days), higher dosages, and haemodialysis also increase risk [56, 57]. According to our literature review, the median age of patients was 67 years, with a predominance of males. The median duration of therapy was consistent with previous studies at 19 days (IQR 12–24). However, therapy dosages varied, with 71% of the population receiving a daptomycin dose of less than 8 mg/kg/day.

Garreau et al. identified a daptomycin AUC24h > 939 mg/h/L as a risk factor for DIEP and suggested as safe an AUC24h target range of 666 to 939 mg/h/L [58]. However, it is necessary to specify that therapeutic drug monitoring (TDM) data are not available in most clinical centres. The TDM was not used in most of the cases included in this review.

Our systematic review found no significant association was noted with past allergies, chronic lung disease, or renal impairment. The initial symptoms often included fever and respiratory issues of varying severity.

Diagnosing DIEP is challenging due to the restrictive nature of ATS criteria. Although increased eosinophils on BAL are essential for a definitive diagnosis, BAL was only performed in 62.2% of cases, with a significant count in just under half. This data demonstrated that it is not always possible to perform a BAL and, when it is, the result is diriment in less than a half of cases. The timing of BAL could affect results, as daptomycin is typically withdrawn quickly once DIEP is suspected, potentially reducing eosinophil counts by the time of the procedure.

The ATS criteria exclude patients who have less than 25% Eosinophils on BAL or who have not undergone the test. In these cases, it is possible to define the case as “possible”.

Regarding the criteria proposed by Phillips, patients who do not present dyspnoea cannot be defined as cases of DIEP. Furthermore, it does not consider patients who cannot undergo BAL or lung biopsy.

In contrast, the Lyon algorithm, which classified 95.5% of reviewed cases as DIEP, offers a more sensitive approach. The advantage of this algorithm is the possibility of identifying such a serious and rapidly evolving pathology more easily and promptly. However, a diagnosis, perhaps incorrect, requires the exclusion of an effective and easy-to-handle drug such as daptomycin. Therefore, it is essential to exclude an alternative diagnosis.

Special attention is necessary for immunocompromised patients, particularly those with haematological conditions, as they may not exhibit typical signs like peripheral eosinophilia or increased eosinophils on BAL [12].

Laboratory findings for the disease are nonspecific, including leukocytosis, eosinophilia, and the detection of Il-5 in bronchoalveolar lavage fluid, which are all associated with acute eosinophilic pneumonia [59].

West et al. observed in a cohort of 330 patients in therapy with daptomycin that elevated peripheral eosinophilia ≥ 5% was a risk factor for daptomycin pulmonary eosinophilia [55]. Peripheral eosinophilia is particularly important for raising suspicion of DIEP, especially since bronchoalveolar lavage (BAL) cannot always be performed. In our data, peripheral eosinophilia was present in 86.5% of reported cases.

Based on the data available, we found that a particular importance should be placed on the radiological findings that, together with the exposure to daptomycin, should represent a red flag for physicians. According to our findings, bilateral and ground-glass opacities, patchy infiltrates, and peripheral predominance are the most frequent radiological characteristics described. The significance of imaging, particularly HRCT, is well-established for diagnosing various forms of acute eosinophilic pneumonia. Common imaging findings include ground glass opacities, interlobular septal thickening, pleural effusion, thickened bronchovascular bundles, air space consolidations, and centrilobular nodules [60].

Regarding therapy, it is obviously essential to quickly discontinue daptomycin. If respiratory function does not improve rapidly or the onset is particularly severe (e.g. need for orotracheal intubation) it may be considered to introduce steroid therapy. Currently, there is no specific dosage or specific criteria for introducing therapy. Patients generally exhibit a rapid response to corticosteroids, with clinical improvement often observed within 48 h and resolution of infiltrates typically occurring within a month. Although the optimal corticosteroid regimen for acute eosinophilic pneumonia has not been definitively established, severe respiratory failure often [61] necessitates high intravenous doses (up to 500 mg/day of methylprednisolone), which can be transitioned to oral prednisone (40–60 mg/day) upon improvement. Oral prednisone is usually maintained for 2–4 weeks, followed by a gradual taper over the subsequent weeks [61].

In several cases, since bacterial pneumonia could not be ruled out, antibiotic therapy was still administered.

The overall percentage of recovery is 78.8% and rapid improvement is a predominant characteristic of the disease. More than half of patients improved after 48 h from daptomycin withdrawal and 87% after 96 h.

The study’s limitations include variable study quality, insufficient data, and a small number of definite cases per ATS criteria.

In conclusion, DIEP is a rare but rapidly progressive adverse event. Early diagnosis and treatment are crucial to avoiding serious consequences. Diagnosis relies on clinical evaluation supported by lab tests and imaging. Daptomycin cessation is fundamental, and corticosteroids may be necessary. Continued research is essential to refine diagnostic criteria for this serious adverse event.

## Data Availability

No datasets were generated or analysed during the current study.

## References

[1] Heidary M, Khosravi AD, Khoshnood S, Nasiri MJ, Soleimani S, Goudarzi M, Daptomycin. J Antimicrob Chemother. 2018;73:1–11. 29059358 10.1093/jac/dkx349

[2] Leone S, Noviello S, Boccia G, De Caro F, Esposito S. Methicillin-resistant Staphylococcus aureus infections: role of daptomycin/β-lactams combination. Le Infez Med [Internet]. 2015;23:99–104. http://www.ncbi.nlm.nih.gov/pubmed/26110289. 26110289

[3] Di Carlo P, D’Alessandro N, Guadagnino G, Bonura C, Mammina C, Lunetta M et al. High dose of trimethoprim-sulfamethoxazole and daptomycin as a therapeutic option for MRSA endocarditis with large vegetation complicated by embolic stroke: a case report and literature review. Le Infez Med [Internet]. 2013;21:45–9. http://www.ncbi.nlm.nih.gov/pubmed/23524901. 23524901

[4] Gidari A, Sabbatini S, Schiaroli E, Perito S, Francisci D, Baldelli F et al. Tedizolid-Rifampicin Combination Prevents Rifampicin-Resistance on in vitro Model of Staphylococcus aureus Mature Biofilm. Front Microbiol [Internet]. 2020;11. https://www.frontiersin.org/article/10.3389/fmicb.2020.02085/full. 10.3389/fmicb.2020.02085 PMC7484889 32983061

[5] Patel JJ, Antony A, Herrera M, Lipchik RJ. Daptomycin-induced acute eosinophilic pneumonia. Wis Med J. 2014;113. 25739164

[6] Kumar S, Acosta-Sanchez I, Rajagopalan N. Daptomycin-induced Acute Eosinophilic Pneumonia. Cureus [Internet]. 2018; https://www.cureus.com/articles/12995-daptomycin-induced-acute-eosinophilic-pneumonia. 10.7759/cureus.2899 PMC6207288 30397557

[7] Hayes D, Anstead MI, Kuhn RJ. Eosinophilic pneumonia induced by daptomycin. J Infect. 2007;54. 10.1016/j.jinf.2006.11.006 17207858

[8] Kim PW, Sorbello AF, Wassel RT, Pham TM, Tonning JM, Nambiar S. Eosinophilic pneumonia in patients treated with daptomycin: review of the literature and US FDA adverse event reporting system reports. Drug Saf. 2012. 10.2165/11597460-000000000-00000 22612850

[9] Phillips J, Cardile AP, Patterson TF, Lewis JS. Daptomycin-induced acute eosinophilic pneumonia: analysis of the current data and illustrative case reports. Scand J Infect Dis. 2013;45. 10.3109/00365548.2013.805427 23826793

[10] Pham TT, Garreau R, Craighero F, Cottin V, Said B, Ben, Goutelle S et al. Seventeen cases of Daptomycin-Induced Eosinophilic Pneumonia in a cohort of patients treated for bone and joint infections: proposal for a New Algorithm. Open Forum Infect Dis. 2022;9. 10.1093/ofid/ofac577 PMC9697587 36447615

[11] Moher D, Shamseer L, Clarke M, Ghersi D, Liberati A, Petticrew M, et al. Preferred reporting items for systematic review and meta-analysis protocols (PRISMA-P) 2015 statement. Rev Esp Nutr Humana y Diet; 2016. 10.1186/2046-4053-4-1 PMC4320440 25554246

[12] Zou J, Rivera Sarti JE, Strasfeld L. Daptomycin-associated pulmonary toxicity sans eosinophilia in a hematopoietic cell transplant recipient with profound leukopenia. Transpl Infect Dis. 2023. 10.1111/tid.14029 36787236

[13] Cobb E, Kimbrough RC, Nugent KM, Phy MP. Organizing pneumonia and pulmonary eosinophilic infiltration associated with daptomycin. Ann Pharmacother. 2007;41. 10.1345/aph.1H372 17374626

[14] Shinde A, Seifi A, DelRe S, Moustafa Hussein WH, Ohebsion J. Daptomycin-induced pulmonary infiltrates with eosinophilia. J Infect. 2009. 10.1016/j.jinf.2008.11.001 19046602

[15] Kakish E, Wiesner AM, Winstead PS, Bensadoun ES. Acute respiratory failure due to daptomycin induced eosinophilic pneumonia. Respir Med CME. 2008;1.

[16] Lal Y, Assimacopoulos AP. Two cases of daptomycin-induced eosinophilic pneumonia and chronic pneumonitis. Clin Infect Dis. 2010;50. 10.1086/650487 20121418

[17] Miller BA, Gray A, Leblanc TW, Sexton DJ, Martin AR, Slama TG. Acute eosinophilic pneumonia secondary to daptomycin: a report of three cases. Clin Infect Dis. 2010;50. 10.1086/652656 20420515

[18] Kalogeropoulos AS, Tsiodras S, Loverdos D, Fanourgiakis P, Skoutelis A. Eosinophilic pneumonia associated with daptomycin: a case report and a review of the literature. J Med Case Rep. 2011;5. 10.1186/1752-1947-5-13 PMC3033840 21241493

[19] Rether C, Conen A, Grossenbacher M, Albrich WC. A rare cause of pulmonary infiltrates one should be aware of: a case of daptomycin-induced acute eosinophilic pneumonia. Infection. 2011;39. 10.1007/s15010-011-0148-y 21717147

[20] Yusuf E, Perrottet N, Orasch C, Borens O, Trampuz A. Daptomycin-associated eosinophilic pneumonia in two patients with prosthetic joint infection. Surg Infect (Larchmt). 2014;15. 10.1089/sur.2013.200 25317960

[21] Hagiya H, Hasegawa K, Asano K, Terasaka T, Kimura K, Nada T et al. Myopathy and eosinophilic pneumonia coincidentally induced by treatment with daptomycin. Intern Med. 2015;54. 10.2169/internalmedicine.54.3397 25758082

[22] Chiu SY, Faust AC, Dand HM. Daptomycin-Induced Eosinophilic Pneumonia treated with Intravenous corticosteroids. J Pharm Pract. 2015;28. 10.1177/0897190014568678 25657193

[23] Wojtaszczyk A, Jankowich M. Dyspnea on daptomycin: eosinophilic pneumonia. R I Med J (2013). 2015;98. 26020264

[24] Roux S, Ferry T, Chidiac C, Valour F. Daptomycin-induced eosinophilic pneumonia. Int J Infect Dis [Internet]. 2015;37:95–6. https://linkinghub.elsevier.com/retrieve/pii/S1201971215001447. 10.1016/j.ijid.2015.06.010 26117342

[25] Montenegro O, Del Campo R, Del Rio JJ, Ambrós Checa A. Acute eosinophilic pneumonia secondary to daptomycin. Enferm Infecc Microbiol Clin. 2016;34. 10.1016/j.eimc.2015.08.010 26530225

[26] Akcaer M, Karakas A, Tok D, Coskun O, Sari S. Eosinophilic pneumonia: daptomycin-induced lung complication. Med Mal Infect. 2016. 10.1016/j.medmal.2016.01.006 26965755

[27] Hatipoglu M, Memis A, Turhan V, Mutluoglu M, Canoglu K. Possible daptomycin-induced acute eosinophilic pneumonia in a patient with diabetic foot infection. Int J Antimicrob Agents. 2016. 10.1016/j.ijantimicag.2016.02.014 27089828

[28] Hirai J, Hagihara M, Haranaga S, Kinjo T, Hashioka H, Kato H et al. Eosinophilic pneumonia caused by daptomycin: six cases from two institutions and a review of the literature. J Infect Chemother. 2017;23. 10.1016/j.jiac.2016.09.001 28003110

[29] Goyal P, Breen MJ, Hountras P, Raj R, Bolon MK. Daptomycin-induced acute eosinophilic pneumonia. Southwest Respir Crit Care Chronicles [Internet]. 2016;4. http://www.pulmonarychronicles.com/index.php/pulmonarychronicles/article/view/239/640.

[30] Nickerson M, Bhargava A, Kale-Pradhan PB. Daptomycin-associated eosinophilic pneumonia with rechallenge: a case report. Int J Clin Pharmacol Ther. 2017;55. 10.5414/CP202952 28406091

[31] Rachid M, Ahmad K, Saunders-Kurban M, Fatima A, Shah A, Nahhas A. Daptomycin-Induced Acute Eosinophilic Pneumonia: Late Onset and Quick Recovery. Case Rep Pulmonol. 2017;2017. 10.1155/2017/8525789 PMC5687141 29225987

[32] Higashi Y, Nakamura S, Tsuji Y, Ogami C, Matsumoto K, Kawago K et al. Daptomycin-induced eosinophilic pneumonia and a review of the published literature. Intern Med. 2018;57. 10.2169/internalmedicine.9010-17 PMC5820046 29093391

[33] Raru Y, Zeid F, Browning S, Saunders E. Two patients with daptomycin induced eosinophilic pneumonia with different presentations and treatment. Respir Med Case Rep. 2018;23. 10.1016/j.rmcr.2017.11.003 PMC5702879 29201637

[34] Alonso GA, Rodríguez IM, Torres JM, Loarte PDV. Daptomycin eosinophilic pneumonia: an adverse effect to be aware of. Rev Esp Quimioter. 2018. PMC6166259 29791122

[35] Basnet S, Tachamo N, Dhital R, Tharu B. Daptomycin associated eosinophilic pneumonia: case report and differential diagnoses. J Community Hosp Intern Med Perspect. 2018;8. 10.1080/20009666.2018.1475188 PMC5998295 29915657

[36] Sharma P, Adhikari J, Merrit A, Khokhar S. Daptomycin Induced Eosinophilic Pneumonia. J Clin Med Ther. 2018;3 No.

[37] Raza A, Arslan A, Atiq MU, Chan V, Patel RK. Unexpected Outcome of Daptomycin-induced Eosinophilic Pneumonia: Rarity Within a Rarity. Cureus. 2019. 10.7759/cureus.6271 PMC6937480 31903307

[38] Storandt MH, Matta A. Acute Eosinophilic Pneumonia: A Rare Complication of Daptomycin Therapy. Cureus. 2020. 10.7759/cureus.6803 PMC7045973 32140361

[39] Somoza-Cano FJ, Makadia A, Cruz-Peralta MP, Zakarna L, Demyda E, Al Armashi AR et al. Acute Eosinophilic Pneumonia Secondary to Daptomycin. Cureus. 2021. 10.7759/cureus.19403 PMC8658045 34926005

[40] Watts A, Toquica Gahona CC, Raj K. Multifocal Pneumonia Amidst the Global COVID-19 Pandemic: A Case of Daptomycin-Induced Eosinophilic Pneumonia. Cureus. 2021. 10.7759/cureus.16002 PMC8318610 34336493

[41] Raman V, Chaudhary I, Shieh S. Eosinophilic pneumonia: a case of daptomycin induced lung injury. J Community Hosp Intern Med Perspect. 2021;11. 10.1080/20009666.2021.1883813 PMC8043520 33889339

[42] Portalatin GM, Chin J-A, Foster B, Perry K, McWilliams C. Daptomycin-Induced Acute Eosinophilic Pneumonia. Cureus [Internet]. 2021; https://www.cureus.com/articles/52135-daptomycin-induced-acute-eosinophilic-pneumonia. 10.7759/cureus.13509 PMC7992914 33786218

[43] Fernández-González R, Díaz López MD, Lorenzo Vizcaya AM, González Noya A. Neumonitis eosinofílica por daptomicina. Med Clin (Barc) [Internet]. 2021;156:148–9. https://linkinghub.elsevier.com/retrieve/pii/S0025775320300397. 10.1016/j.medcli.2019.10.015 32098706

[44] Fujii E, Arita T, Uejima T, Matsuhama M, Iida M, Inoue T et al. Blood culture-negative prosthetic valve endocarditis and daptomycin-associated eosinophilic pneumonia: a case report. J Cardiol Cases. 2022;25. 10.1016/j.jccase.2021.12.008 PMC9169011 35685267

[45] Valaiyapathi R, Wu MS, McGregor A. Ground glass opacities are not always COVID-19: a case of acute eosinophilic pneumonitis caused by daptomycin. Lancet. 2022. 10.1016/S0140-6736(22)00009-5 PMC8758180 35033220

[46] Eckhardt LGJ, Kelley JL, Maes D. Challenges in managing a multifactorial eosinophilic pneumonia: daptomycin vs strongyloidiasis case report. BMC Infect Dis. 2022;22. 10.1186/s12879-022-07852-y PMC9682830 36418971

[47] Abd Algayoum R, Elsherif A, Khan ZH, Roman G. Daptomycin-Induced Eosinophilic Pneumonia Mimicking Multifocal Pneumonia. Cureus. 2022. 10.7759/cureus.30521 PMC9675429 36415446

[48] Gerts N, Nathan R, Tabet A, Merchant M, Takher J. A Case of Daptomycin-Induced Eosinophilic Pneumonia. Ann Intern Med Clin Cases [Internet]. 2023;2. https://www.acpjournals.org/doi/10.7326/aimcc.2022.0827.

[49] Sharma S, Rojas H, Spano C, George-Varghese B, Liu T. Acute Eosinophilic Pneumonia presenting as altered Mental Status. J Emerg Med. 2023;64. 10.1016/j.jemermed.2023.02.020 37002159

[50] Patel YI, Natarajan S, Ramakrishna S, Ochieng P. Daptomycin-Induced Pulmonary Toxicity: A Case Series. Cureus. 2023. 10.7759/cureus.39613 PMC10299848 37384075

[51] Arcalas C-J, Artola R, Hanna ME, Li H, Levy S, Kalyatanda GS. An Unexpected Turn of Events in a Patient With Mitral Valve Endocarditis. Cureus. 2023. 10.7759/cureus.42121 PMC10436997 37602086

[52] Carbone RG, Puppo F, Mattar E, Roden AC, Hirani N. Acute and chronic eosinophilic pneumonia: an overview. Front Med [Internet]. 2024;11. https://www.frontiersin.org/articles/10.3389/fmed.2024.1355247/full. 10.3389/fmed.2024.1355247 PMC11070545 38711783

[53] Uppal P, LaPlante KL, Gaitanis MM, Jankowich MD, Ward KE. Daptomycin-induced eosinophilic pneumonia - a systematic review. Antimicrob Resist Infect Control 2016. 10.1186/s13756-016-0158-8 PMC5153904 27999664

[54] Soldevila-Boixader L, Villanueva B, Ulldemolins M, Benavent E, Padulles A, Ribera A et al. Risk Factors of Daptomycin-Induced Eosinophilic Pneumonia in a Population with Osteoarticular Infection. Antibiotics [Internet]. 2021;10:446. https://www.mdpi.com/2079-6382/10/4/446. 10.3390/antibiotics10040446 PMC8071505 33923382

[55] West KA, Sheeti A, Tamura MacKay K, Forrest GN. Eosinophilic Syndromes Associated With Daptomycin Use: Re-exposure Hypersensitivity Pneumonitis and Prior Peripheral Eosinophilia. Open Forum Infect Dis [Internet]. 2022;9. https://academic.oup.com/ofid/article/doi/10.1093/ofid/ofac065/6549660. 10.1093/ofid/ofac065 PMC8926001 35308486

[56] Okada N, Niimura T, Saisyo A, Kawaguchi Y, Ishizawa K, Kitahara T. Pharmacovigilance Study on Eosinophilic Pneumonia Induced by Anti-MRSA Agents: Analysis Based on the FDA Adverse Event Reporting System. Open Forum Infect Dis [Internet]. 2023;10. https://academic.oup.com/ofid/article/doi/10.1093/ofid/ofad414/7235580. 10.1093/ofid/ofad414 PMC10438871 37601729

[57] Ishikawa K, Matsuo T, Tsuda Y, Rahman M, Uehara Y, Mori N. Factors Associated with Daptomycin-Induced Eosinophilic Pneumonia. Antibiotics [Internet]. 2022;11:254. https://www.mdpi.com/2079-6382/11/2/254. 10.3390/antibiotics11020254 PMC8868239 35203855

[58] Garreau R, Pham T-T, Bourguignon L, Millet A, Parant F, Bussy D et al. Daptomycin Exposure as a Risk Factor for Daptomycin-Induced Eosinophilic Pneumonia and Muscular Toxicity. Clin Infect Dis [Internet]. 2023;77:1372–80. https://academic.oup.com/cid/article/77/10/1372/7226491. 10.1093/cid/ciad386 37467019

[59] Allen JN, Liao Z, Wewers MD, Altenberger EA, Moore SA, Allen ED. Detection of IL-5 and IL-1 receptor antagonist in bronchoalveolar lavage fluid in acute eosinophilic pneumonia. J Allergy Clin Immunol [Internet]. 1996;97:1366–74. https://linkinghub.elsevier.com/retrieve/pii/S0091674996702063. 10.1016/s0091-6749(96)70206-3 8648034

[60] Daimon T, Johkoh T, Sumikawa H, Honda O, Fujimoto K, Koga T et al. Acute eosinophilic pneumonia: Thin-section CT findings in 29 patients. Eur J Radiol [Internet]. 2008;65:462–7. https://linkinghub.elsevier.com/retrieve/pii/S0720048X0700201X. 10.1016/j.ejrad.2007.04.012 17537607

[61] Rhee CK, Min KH, Yim NY, Lee JE, Lee NR, Chung MP et al. Clinical characteristics and corticosteroid treatment of acute eosinophilic pneumonia. Eur Respir J [Internet]. 2013;41:402–9. http://erj.ersjournals.com/lookup/doi/10.1183/09031936.00221811. 10.1183/09031936.00221811 22599359

